# “What if” should precede “whether” and “how” in the social conversation around human germline gene editing

**DOI:** 10.1007/s12687-023-00652-0

**Published:** 2023-06-16

**Authors:** Diewertje Houtman, Wendy Geuverink, Isabel Rosalie Arianne Retel Helmrich, Boy Vijlbrief, Martina Cornel, Sam Riedijk

**Affiliations:** 1 Department of Clinical Genetics, Erasmus Medical Center, Rotterdam, The Netherlands; 2 Human Genetics, Amsterdam UMC, Vrije Universiteit Amsterdam, Amsterdam, The Netherlands; 3 Amsterdam Reproduction and Development Research Institute, Amsterdam, The Netherlands; 4 Amsterdam Public Health Research Institute, Amsterdam, The Netherlands

**Keywords:** Human germline gene editing, Public engagement, Futures literacy, Societal alignment

## Abstract

Given the potential large ethical and societal implications of human germline gene editing (HGGE) the urgent need for public and stakeholder engagement (PSE) has been repeatedly expressed. In this short communication, we aim to provide directions for broad and inclusive PSE by emphasizing the importance of futures literacy, which is a skill to imagine diverse and multiple futures and to use these as lenses to look at the present anew. By first addressing “what if” questions in PSE, different futures come into focus and limitations that arise when starting with the “whether” or “how” questions about HGGE can be avoided. Futures literacy can also aid in the goal of societal alignment, as “what if” questions can be answered in many different ways, thereby opening up the conversation to explore a multitude of values and needs of various publics. Broad and inclusive PSE on HGGE starts with asking the right questions.

## Towards societal alignment

Decisions about whether or not to pursue human germline gene editing (HGGE; Table 1) should be aligned with the values and needs within society, as it raises unanswered moral and societal questions. Some of these questions include concerns about, but are not limited to, issues related to respect for human life and dignity, the moral status of the human embryo, inequalities in and between societies, and the overall impact on future children who did not provide consent to alter their genes (Almeida & Ranisch 2022; Howard et al. 2018; Ormond et al. 2017). Furthermore, the use of HGGE could reduce the acceptance of people with disabilities due to the so-called “expressivist objection”, which holds that the use of these technologies conveys and perpetuates negative perceptions about certain disabling conditions and the people currently living with these conditions (Boardman 2020; Hoffman‐Andrews et al. 2019). Given the potential large ethical and societal implications, these decisions should not be left to scientists alone (Almeida and Ranisch 2022; Baylis 2017). Therefore, the urgent need for public and stakeholder engagement (PSE) on HGGE has repeatedly been expressed by various authoritative national and international bodies and committees (Chan et al. 2015; Lander et al. 2019; National Academies of Sciences 2017; National Academy of Medicine 2020; Nuffield Council on Bioethics 2018; WHO Expert Advisory Committee on Developing Global Standards for Governance and Oversight of Human Genome Editing, 2021).

**Table 1:** Description of human germline gene editing

**What is human germline gene editing and how is it different from somatic gene editing?**
“Somatic genome editing refers to the alteration of cells that cannot contribute to gamete formation and thus cannot be passed on from the individual to offspring. In contrast, **germline genome editing** […] refers to genome editing that occurs in a germ cell or embryo and results in changes that are theoretically present in all cells of the embryo and that could also potentially be passed from the modified individual to offspring. In theory, modification of gamete-producing cells at any point in development could permit this. Because human germline genome editing has potential effects on both the treated individual and subsequent generations of persons, it entails ethical considerations beyond those of somatic genome modification.”—Ormond et al. (2017)

PSE has the potential to enable policy making that is informed by the values and needs of society at large (Scheufele et al. 2021). Across different disciplines, there are examples of PSE efforts that succeeded to gain insights into the values and needs of society at large which subsequently informed policy-making (McKee 2018). That said, while PSE efforts that are issued by governmental policymakers generally feed into policymaking, the output of PSE initiated by researchers does not self-evidently find its way into policy-making (Pieczka and Escobar 2013; van den Bongaardt 2018). To be effective and enable policy-making, PSE must be comprehensive, transparent, inclusive, methodologically sound and accountable (Iltis et al. 2021). In working towards societal alignment, we will be ready to make decisions when we are fully informed about the shared and conflicting values and needs that are at stake. To map this large palette of values and needs, a wide diversity of publics needs to be engaged. In this short communication, we aim to provide directions for broad and inclusive PSE by emphasizing the importance of future narratives when deliberating on HGGE.

## Inclusive public and stakeholder engagement

Inclusivity is described as one of the ideals for effective PSE in developing HGGE policies (Iltis et al. 2021) and the WHO’s Expert Advisory Committee explicitly calls for inclusive dialogue on the future of human genome editing (WHO Expert Advisory Committee on Developing Global Standards for Governance and Oversight of Human Genome Editing, 2021). This asks for specific efforts to include groups that are underrepresented or even missing completely, because in PSE, we typically reach participants who are highly educated, knowledgeable on the topic, predominantly white, affluent, and have relatively high trust in science (Boardman 2020; Humm et al. 2020). Missing the voices of underrepresented groups poses a threat to the development of societally aligned HGGE governance and regulation. Prior research shows that emotional factors (e.g. fear and habitual distance) play an important role in excluding underrepresented people (Humm et al. 2020). The exclusion of underrepresented groups may be influenced by how the conversation on HGGE is framed in PSE. By focusing solely on the conditions under which HGGE can proceed, we risk exclusion of those who hold fundamental objections against HGGE or those who have yet to make up their mind for various reasons (Iltis et al. 2021). Thus, inclusive PSE for HGGE requires reflection on the value and purpose of the PSE effort itself.

## The social conversation around human germline gene editing

Bucchi and Trench (2021) defined science communication as the social conversation around science. This definition does not limit science communication to initiatives organized by people from scientific communities talking to the public. It includes all types and forms of conversation between different people and in different places, both online and offline. To increase the democratic value of PSE, efforts should be made to capture and feed instead of steer the societal discourse regarding HGGE. As (social) scientists, we need to understand that the questions we ask, influence the answers that are given (Smit and Hessels 2021). If we only ask “how” HGGE should be applied, we will not hear all relevant considerations regarding “whether” we should apply HGGE (Baylis 2019b). However, the “whether” question asks for deliberation. In striving for societal alignment, the values and needs of society need to be made explicit. To identify our values and needs for the future, “what if” questions should be addressed first.

## Questions of “what if”

**Fig. 1:**
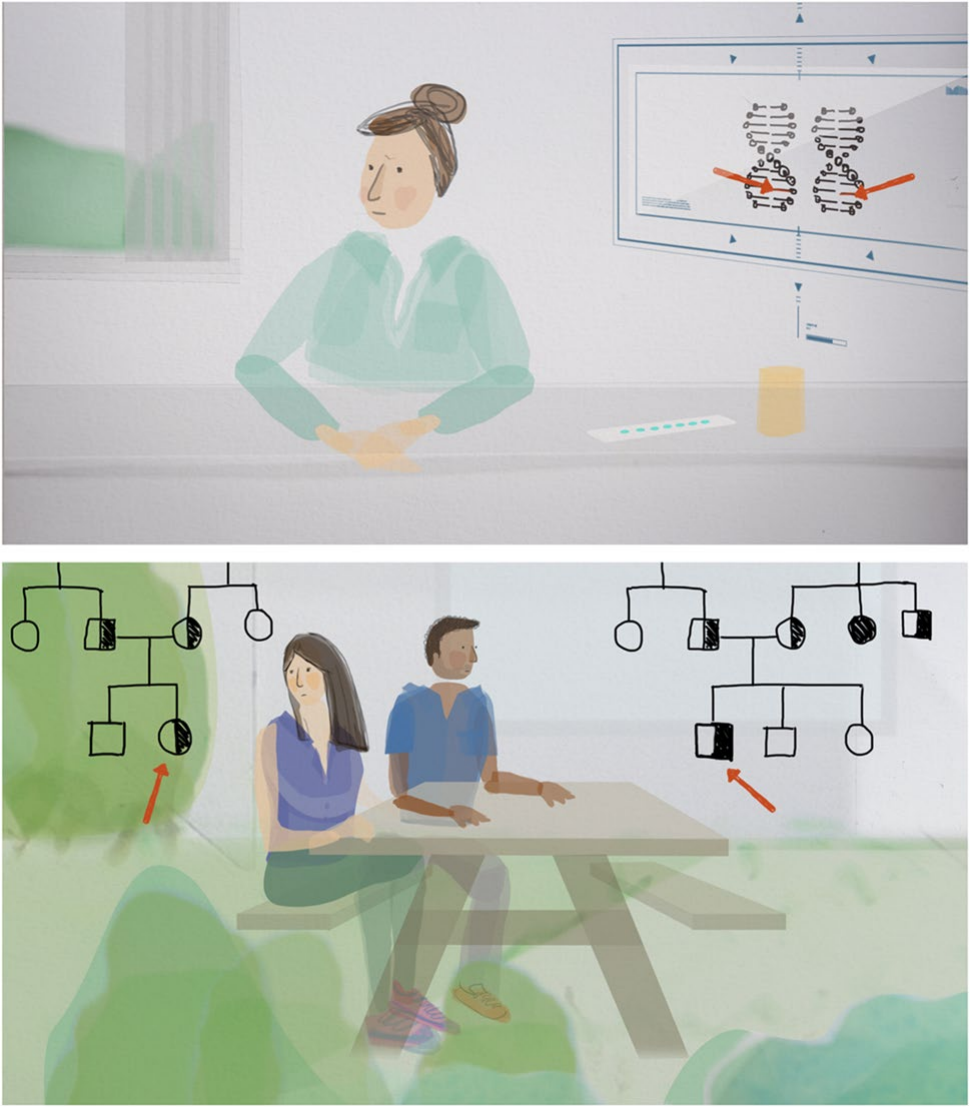
Stills from animations by Zaou Vaughan that were developed within the DNA dialogue project. *Note.* The animations depict three different future narratives of the world in 2039, in which couples with a childwish consider their reproductive options. The futures are titled as follows: (1) The Netherlands say no to genetic modification of embryos, (2) Caring for your baby starts before conception and (3) Equality begins in the genes. For more information about the future narratives and to watch the full animations, see https://www.rathenau.nl/en/gezondheid/discussing-modification-heritable-dna-embryos

‘Globally’, as Françoise Baylis writes, ‘the political consensus on heritable human genome editing—such as it is—inclines toward an outright ban, and if not a ban, at least a moratorium’ (Baylis 2019a). Although the scope and means of these regulations and ‘bans’ vary widely internationally, they still provide the opportunity to discuss various futures and to empower people to form their opinions. Opinion-forming can be supported by future narratives about what decisions on HGGE could look like. This skill to imagine diverse and multiple futures and to use these as lenses to look at the present anew, is called futures literacy (Damhof et al. 2020; Miller 2018). Future narratives are especially helpful when it comes to potentially disruptive and relatively unfamiliar technologies, such as HGGE, where the future impact is still difficult to foresee, yet now is the time to make decisions and influence how the technology will be governed (Collingridge 1982). In the Dutch DNA dialogue (Table 2), a broad societal dialogue to inquire about the views of the Dutch society on HGGE, animations were used to visualize future narratives (Fig. 1) (van Baalen et al. 2021). The animated future narratives each sketched a different society based on three strategies of applying or not applying HGGE. During the PSE, moderators use these narratives as conversations starters while emphasizing that these scenarios are not inevitable or “the truth”. As such, the future narratives provided guidance for reflection and discussion about HGGE during the dialogues. Using various scenarios that focus on different dilemmas and that show a multitude of perspectives might avoid scenario simplification and unbalanced steering. Futures literacy can aid in the goal of creating societal alignment as questions of “what if” stimulate imagination, thereby opening up the conversation to explore the values and needs of various publics (Ribeiro et al. 2018).Join the conversation about modifying

**Table 2:**
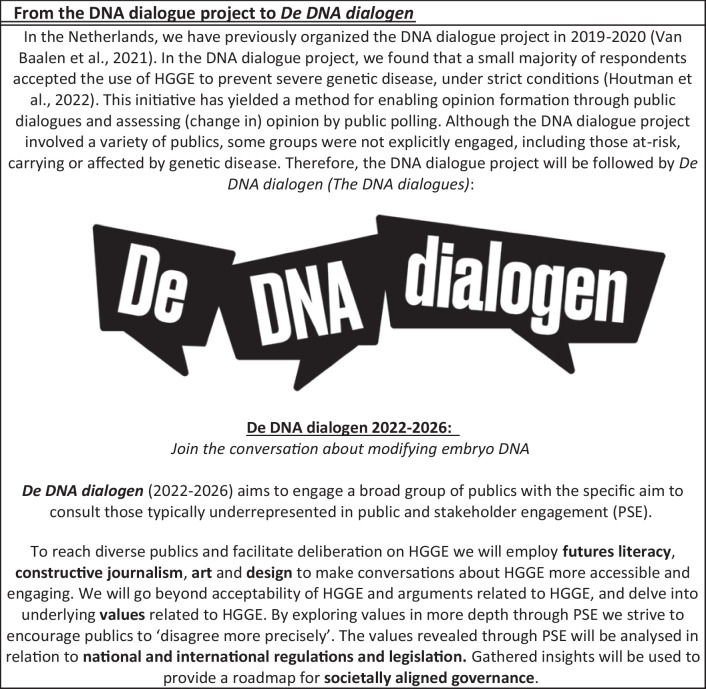
From the DNA dialogue project to De DNA dialogen

## The questions of “whether” and “how”

The “what if” questions require time for the public to reflect, slow down, use imagination and think beyond their current lived experiences. They allow the public to think about the ways in which HGGE could improve their lives and the lives of their children, as well as which futures would be undesirable and inconsistent with their values. This speculative “if yes, then how” question should not be confused with the question of “how” HGGE should be implemented. Therefore, it is important to be transparent about this distinction at the outset of PSE. The questions “whether” and “how” provide valuable insight in how practice and policy around HGGE should be organized, which will become relevant once the scenarios of what our society may become are deliberated on. Until then, there is a widely shared responsibility to enable this deliberation and opinion formation (Houtman et al. 2021). Scientists, scientific societies and policymakers who seek to engage the public should lead by example by making the missing voices explicit and by making room for people who believe the technology should never be used. Broad and inclusive PSE by imagining future narratives is a prerequisite for policy-making in which all voices have been heard and considered and which is aligned with a wide range of societal values and needs.

## Conclusion

Broad and inclusive social conversation around HGGE starts with asking the right questions. By focusing on questions of “what if” before “whether” and “how” in PSE we can open up the conversation by creating various future narratives. Consequently, we reduce directivity and framing as initiators of PSE, and increase inclusiveness as this approach does not exclude in advance those who hold fundamental objections against HGGE or those who have yet to make up their mind for various reasons. Actual participation in PSE by the aforementioned groups is something that should be evaluated and monitored continuously. Asking “what if” will surface the values and needs that are at stake, which can then be taken into consideration when the questions of “whether” and possibly “how” are deliberated on.
